# A machine learning photon detection algorithm for coherent x-ray ultrafast fluctuation analysis

**DOI:** 10.1063/4.0000161

**Published:** 2022-10-17

**Authors:** Sathya R. Chitturi, Nicolas G. Burdet, Youssef Nashed, Daniel Ratner, Aashwin Mishra, T. J. Lane, Matthew Seaberg, Vincent Esposito, Chun Hong Yoon, Mike Dunne, Joshua J. Turner

**Affiliations:** 1 Department of Materials Science and Engineering, Stanford University, Stanford, California 94305, USA; 2 SLAC National Accelerator Laboratory, Menlo Park, California 94025, USA; 3 Stanford Institute for Materials and Energy Sciences, Stanford, California 94305, USA; 4 Deutsches Elektronen-Synchrotron, Hamburg, Germany

## Abstract

X-ray free electron laser experiments have brought unique capabilities and opened new directions in research, such as creating new states of matter or directly measuring atomic motion. One such area is the ability to use finely spaced sets of coherent x-ray pulses to be compared after scattering from a dynamic system at different times. This enables the study of fluctuations in many-body quantum systems at the level of the ultrafast pulse durations, but this method has been limited to a select number of examples and required complex and advanced analytical tools. By applying a new methodology to this problem, we have made qualitative advances in three separate areas that will likely also find application to new fields. As compared to the “droplet-type” models, which typically are used to estimate the photon distributions on pixelated detectors to obtain the coherent x-ray speckle patterns, our algorithm achieves an order of magnitude speedup on CPU hardware and two orders of magnitude improvement on GPU hardware. We also find that it retains accuracy in low-contrast conditions, which is the typical regime for many experiments in structural dynamics. Finally, it can predict photon distributions in high average-intensity applications, a regime which up until now has not been accessible. Our artificial intelligence-assisted algorithm will enable a wider adoption of x-ray coherence spectroscopies, by both automating previously challenging analyses and enabling new experiments that were not otherwise feasible without the developments described in this work.

## I. INTRODUCTION

The construction and operation of x-ray free electron lasers (XFELs)^1–5^ have enabled a great leap toward deeper understanding of a diverse area of scientific research areas,^6^ including planetary science,^7^ astrophysics,^8^ medicine,^9^ and molecular chemistry.^10^ With the unprecedented brightness, short pulse duration, and x-ray wavelengths, new states of matter can be created and studied,^11^ while dynamics can be monitored, and now controlled, on ultrafast timescales.^12^

With the start of high repetition rate next-generation light sources, methods which have so far been challenging may become feasible, such as resonant inelastic x-ray scattering at high time- and spectra-resolution^13^ and x-ray photoemission spectroscopy.^14^ One such example is x-ray photon correlation spectroscopy (XPCS),^15,16^ which uses the spatial coherence of the x-ray beam to produce a scattering “fingerprint” of the sample. This fingerprint, or speckle pattern, can be correlated in time to directly observe equilibrium dynamics of a given system. This information on the thermal fluctuations can be related back to both the energetics and the interactions in the system. This is typically measured by calculating the intensity–intensity autocorrelation function $g^{\left(2\right)}(q,t)$ and extracting the intermediate scattering function *S*(*q*, *t*) using the following equation:

$$g^{\left(2\right)}(q,t)=1+AS{\left(q,t\right)}^{2},$$ (1)

where *A* is commonly known as the contrast factor and accounts for properties of the beam. This allows the time correlation to be related back to the physical properties of the system being studied.

Another benefit of these new machines is in their ability to produce finely spaced x-ray pulses with controllable delay, using x-ray optics^17^ or special modes of the accelerator.^18^ These pulses enable studies of spontaneous fluctuations at orders of magnitude faster timescales than what is possible using XPCS at x-ray synchrotron facilities, with one key area of application being emergent phenomena in quantum materials. We refer to this multi-pulse adding technique here as x-ray photon fluctuation spectroscopy (XPFS).^19^ This is a unique tool which differs from traditional pump–probe spectroscopy, which detects the relaxation from a non-equilibrium state, by instituting more of a “probe–probe” method, where fluctuations in the equilibrium state can be measured directly by comparing how the system changes between probe pulses. Here, one adds the pulses which are too close together in time to be readout by the detector^20^ and uses statistics of the coherent speckle^21^ to compute the fluctuation spectra using the contrast,^22,23^ i.e., the fast dynamical information of the system can be distinguished by studying single photon fluctuations. Even with the massive amount of photons per pulse, three things typically result in a single photon detection process: the decrease in intensity after the scattering process on a single pulse basis, the short pulse duration, and the sometimes reduced intensity required to ensure excitations are not produced in the sample.

In principle, if the discrete distribution of photon counts over the detector can be accurately measured and enough samples averaged, it is possible to determine the dynamical evolution the sample by computing the speckle contrast C(q,t) as a function of delay-time *t* and momentum transfer *q*. The contrast is obtained by fitting a negative binomial distribution^24^ parameterized by M≡M(q,t) = $\frac{1}{C^{2}\left(q,t\right)}$ and the average number of photons per pixel $\bar{k}$, i.e., $P\left(k;\bar{k},M\right)$:

$$P(k,\bar{k},M)=\frac{\Gamma (k+M)}{k!\Gamma (M)}{\left(\frac{\bar{k}}{\bar{k}+M}\right)}^{k}{\left(\frac{M}{\bar{k}+M}\right)}^{M}.$$ (2)

Fitting this negative binomial distribution requires the extraction of photon counts from raw detector images and works fairly well in the hard x-ray regime and for large pixel size detectors.^24–26^ In cases where the pixels are small, or the energy of the x-rays is much lower, this process can involve additional obstacles. One challenge is the point spread function of a single photon can spread non-uniformly over many pixels. This is especially true in the soft x-ray regime, where there can be a large variability in the charge cloud size owing to variable diffusion lengths within a pixel and low signal to noise ratios. These effects have recently been shown to be corrected by a variational droplet model called the Gaussian Greed Guess (GGG) droplet model,^27^ which can fit the large variation in charge cloud radii to produce discrete images where each pixel contains the number of corresponding photons.

While droplet-type models have been largely successful, there is a need to increase the speed of these computational models as well as to handle common scenarios, such as low signal-to-noise. A few works have employed machine learning techniques to address some of these outstanding challenges. For instance, the use of convolutional neural networks to analyze XPCS data for well-resolved speckles has showed the denoising approaches can achieve significantly better signal-to-noise statistics as well as estimations of key parameters of interest.^28–30^ Previous work has also considered the single-photon analysis for hard x-ray detectors using machine learning. One approach^31^ has been to use a Tensorflow computational graph with hand-crafted convolutional masks derived from an in-depth study of photon physics at semiconductor junctions.^32^ This implementation is extremely fast, but does not apply to regimes where there may be a large number of photons per droplet. Another method^33^ proposes a feed-forward neural network architecture, based on a sliding prediction of 5 × 5 regions of the input image. This was proposed for the photon map prediction task and is shown to be applicable for hard x-ray, low count rate experiments. However, additional factors such as noise, low photon energies, and insufficient signal-to-noise ratios can cause limitations in this methodology and thus obscure scientific results. Furthermore, in cases where a higher intensity can be measured, the charge clouds can quickly coalesce, making this problem intractable.

In this work, we expand the applicability of this ultrafast method by demonstrating robust single-shot prediction using an artificial intelligence (AI)-assisted algorithm in the soft x-ray regime for data with relatively high average count-rates and significant charge sharing. This is carried out using a fully convolutional neural network (CNN) architecture,^34^ which we compare against the GGG method, currently the best algorithm for soft x-ray analysis using small pixel-size detectors.^27^ We find that we are able to access a new phase space of measurement parameters that, until now, has not been accessible in structural dynamics studies using the XPFS technique. Our algorithm enables a two order of magnitude speedup on appropriate hardware, is relatively accurate for low contrast cases, and is stable at higher intensities than the GGG algorithm. We first describe the machine learning model and simulator used to train it, specifying the architecture, how the model is trained, and the evaluation metric used. This is followed by our main results, and the three areas which were shown to return excellent results relative to the current state-of-the-art models. Finally, we end with a discussion of uncertainty quantification, and how one can judge the error for different models.

## II. MODELING AND ANALYSIS APPROACH

### A. Simulator description

One key issue in the development of supervised machine learning algorithms is a robust simulator which can adequately describe the data. To describe the simulator here, we denote an input XPFS frame as $x_{i}\in {\mathbb{R}}^{90\times 90}$ and the corresponding output photon map as $p_{i}\in {\mathbb{R}}^{30\times 30}$. The 3 × 3 reduction in dimensionality between *x_i_* and *p_i_* is used to mimic the speckle oversampling factor that is typically used in Linac Coherent Light Source (LCLS) experiments. The final calculations are performed on a 30 × 30 image to allow for the proper photon events to be expressed per speckle.

**FIG. 1.**
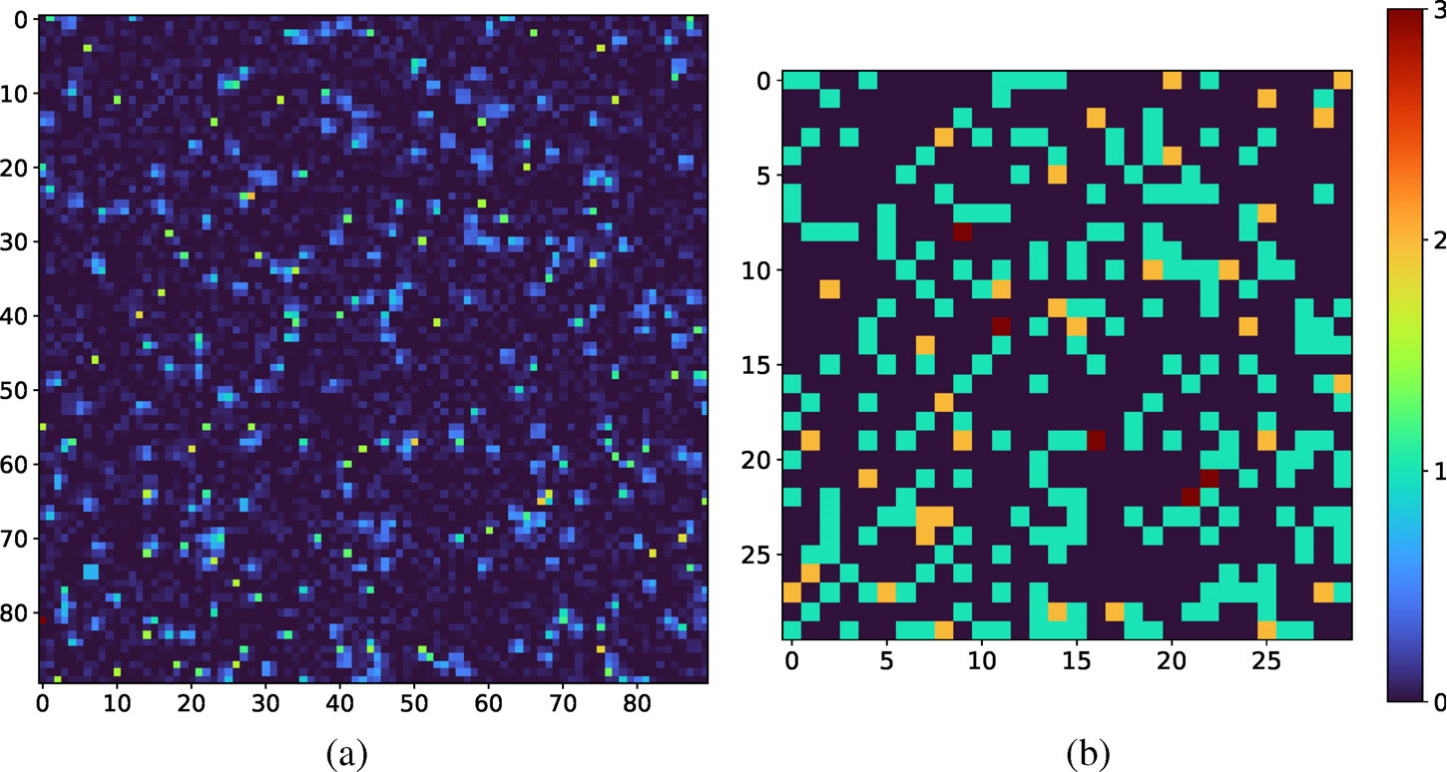
(a) An example of an XPFS detector image over a 90 × 90 pixelated detectors. (b) Corresponding image of the photon map produced by the simulator for the detector image, plotted as the photon distribution per speckle.

The detector images and corresponding photon maps are simulated according to the exact parameters described in Ref. 27, which were tuned to mimic a previous experiment by matching the overall pixel and droplet histogram. To simulate ground truth photon maps, the following ranges were used: $\bar{k}$ ∈ (0.025, 2.0) and C(q,t) ∈ (0.1, 1.0). The relevant detector parameters are the probabilities (*w_i_*) and sizes (*σ_G_*) of the photon charge clouds, the variance of the zero-mean Gaussian background detector noise (*σ_N_*) and the total number of analog-to-digital units (ADUs) per photon. These parameter values are reproduced below in Table I and an example of a detector image/photon map pair is shown in Fig. 1. For proper comparison, we used the Gaussian Greedy Guess (GGG) algorithm with relevant parameters which were optimized for these specific simulation parameters described above.^27^

**TABLE I.** Simulation parameters used to generate detector images based on previous XPFS data collected by Seaberg *et al.* at the iron *L*-edge for magnetic scattering.^35^

Number of pixels per droplet	1	2	3	4	5	6
*σ_G_*	0.10	0.25	0.35	0.45	0.55	0.60
*w_i_*	0.25	0.15	0.1	0.3	0.15	0.05
Photon ADU = 340	*σ_N_* = 15 ADU					

### B. Model architecture

In this problem, we seek a supervised machine learning model which learns the functional mapping $f:x_{i}\mapsto p_{i}$, from *N_f_* paired simulated data points ( $x_{i=1:N_{f}},p_{i=1:N_{f}}$). Specifically, given a raw detector image, the output of the machine learning model is the predicted photon map. Later in the uncertainty quantification section, we add to this approach by using an ensemble of machine learning models. In this case, the output prediction is a median photon map and a standard deviation photon map (Sec. III B).

**FIG. 2.**
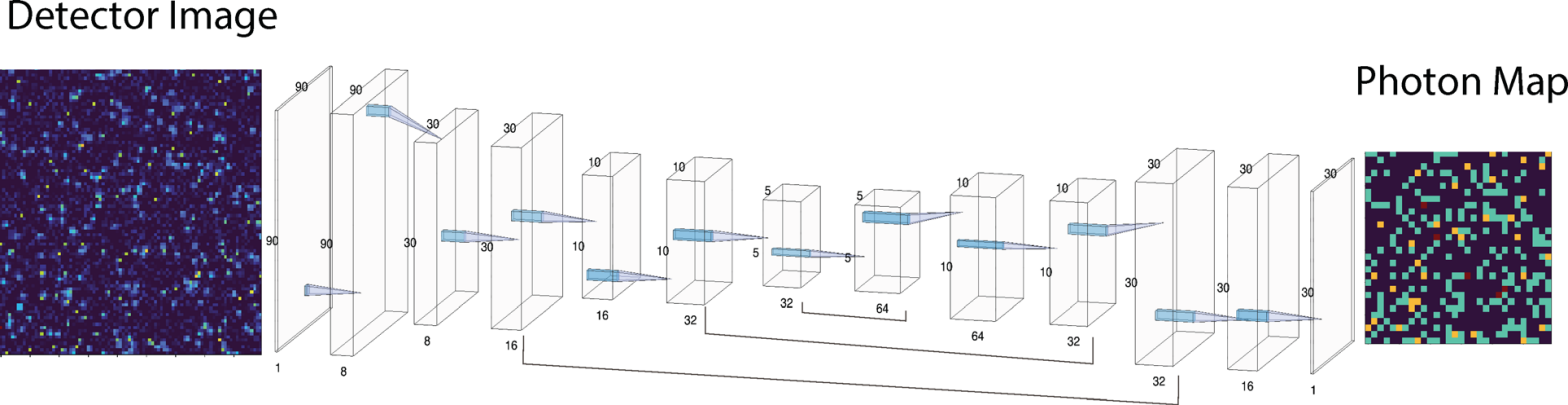
A schematic for the U-Net neural network developed here for single photon counting detection. The input is given by the 90 × 90 detector image with output shown of the resultant 30 × 30 speckle photon map. Convolutional block layers are shown between the input and output images using the NN-SVG package.^38^

In this case, the specific functional mapping was chosen to be a U-Net neural network (Fig. 2), a fully convolutional autoencoder architecture, which is characterized by having skip-connections between different layers of resolution and has been shown to perform well on various image segmentation tasks.^36^ In the schematic in Fig. 2, the architecture is outlined via successive “convolutional blocks.” Each such convolutional block consists of two convolutional layers sequentially applied to the input. After each convolution, we utilize batch normalization^37^ to ensure robust optimization, followed by a Rectified Linear Unit (ReLU) activation. The choice for using a fully convolutional architecture was motivated by the insight that the important features are expected to be local. The reason for this claim is that the charge from a photon will only be able to diffuse to a small number of neighboring pixels owing to large energy barriers across pixel boundaries.^32^ Therefore, to make a prediction for a given pixel, only a small subset of the image is needed (generally no further than a pixel's second nearest neighbor). Furthermore, the point-spread-function of the photon does not really depend on the pixel location on the detector. In addition, the fully convolutional architecture allows for variable input sizes and therefore can be used to analyze larger regions of interest (ROIs) on a given detector.

### C. Training details and validation metrics

To train the model, we use the Frobenius norm between the predicted photon maps ( ${\hat{P}}_{i}$) and the true photon maps (*P_i_*). This loss function measures the mean squared deviation between the predicted photon map and the true photon map, where the average is taken both within a given frame (which contains *N_p_* pixels) and between frames (*N_f_*) in the dataset. Here, we note that various loss functions were considered for this problem during our numerical experiments. In particular, we had experimented with the negative log-likelihood loss in order to directly predict a mean and standard deviation for each pixel. This would conform to the model referred to as a probabilistic neural network, which does not make an assumption of homoscedasticity. However, we found that the model optimization did not converge well. We also observed a similar outcome when using an evidential deep learning approach.^39^ Loss functions such as the L1, Huber, and Frobenius norms gave good predictions in terms of validation performance for the contrast prediction. Additionally, we found that framing the problem as a multi-class classification problem and using the categorical cross-entropy loss gave comparable results. However, ultimately, we opted against the latter procedure as it required pre-specifying the total number of classes (i.e., the maximum photon count) for the experiment and therefore imposed an un-physical clipping operation for the data,

$$L\left(P,\hat{P}\right)=\sum_{i=1}^{N_{f}}\sum_{j=1}^{N_{p}}||P_{i,j}-{\hat{P}}_{i,j}|{|}_{2}^{2}.$$ (3)

The U-Net model is trained by minimizing $L\left(P,\hat{P}\right)$ with respect to the model parameters. To train the neural network, we use the following hyperparameters: Adaptive Moment Estimation (ADAM) algorithm for optimization ( $\beta_{1}=0.9,\;\beta_{2}=0.999$),^40^ batch size = 128, learning rate = 0.001, and batch normalization. We used NVIDIA A100 GPU hardware with the Keras API.^41^ In addition, the input simulated detector images were normalized by the photon ADU for the data (340 ADU).

We consider two primary regimes of count rates ( $\bar{k}$): low $\bar{k}$, which consists of data in the range (0.025, 0.2) and full-range $\bar{k}$, which consists of data in the range (0.025, 2.0). The first range is representative of data which can be analyzed by the GGG model in experiments, while the second range encompasses the range of data previously collected but not compatible with droplet-type models. We train models for both regimes and denote them the low $\bar{k}$ model and the full-range $\bar{k}$ model, respectively. Note, for some analyses we use the term high $\bar{k}$, which considers data only in the range (0.2, 2.0).

For the low $\bar{k}$ data, 100 000 training data points were simulated based on the detector parameters in Table I and uniformly selecting $\bar{k}$ in range (0.025, 0.2) and C(q,t) in the range (0.1, 1.0). Concretely, each frame in the dataset samples a random value for $\bar{k}$ in range (0.025, 0.2) and C(q,t) in range (0.1, 1.0). Then the photon populations are determined by sampling the negative binomial distribution with the corresponding $\bar{k}$ and C(q,t) as parameters. Once the photon populations are determined, they are randomly assigned to pixels within a given frame. Therefore, the underlying photon distributions will differ frame to frame. After the positions are determined, charge smearing and noise is added according to Table I.

For the full-range $\bar{k}$ analysis, 300 000 data points were used for training with an equal proportion of data points coming from $\bar{k}$ ∈ (0.025, 0.2), (1.0, 2.0), and (0.025, 2.0), respectively, with the C(q,t) randomly chosen from the range (0.1, 1.0). This process can be conceptualized as for a given frame, choosing a specific range with probability 1/3 and then choosing a specific $\bar{k}$ value from that range. This approach was chosen to ensure good coverage within the full range of $\bar{k}$ values.

For the validation and test datasets, a slightly different procedure was chosen. Since our ultimate metric is contrast (and not necessarily the mean squared deviation of the predicted photon map), our validation and test data are carefully chosen to reflect this. In general, the contrast is only really determinable using thousands of frames as a large amount of data are needed to perform distribution fitting. Therefore, for the low $\bar{k}$ data [(0.025, 0.2)], we actually have 18 validation and test sets of sizes 5000 and 2000, respectively. In each dataset, the $\bar{k}$ ranges are the same as the training set (0.025, 0.2) and are chosen uniformly, for each frame, as above. However, in this case, for each dataset we fix a specific and different value for the contrast. For example, dataset 1 has a contrast of 0.05 and dataset 18 has a contrast of 0.95. In the full-range $\bar{k}$ data, we actually have 54 (18 × 3) validation and testing sets of sizes 5000 and 2000, respectively. The 3 in this case refers to datasets simulated with $\bar{k}$ in the ranges (0.025, 0.2), (1.0, 2.0), and (0.025, 2.0). The reason for having separate $\bar{k}$ regions was to be able to select models only if they performed well in all three ranges.

Using these predicted contrasts for different contrast levels, we form a contrast–contrast parity plot for the ground truth contrast vs predicted contrast. To select between competing trained models, the optimal neural network was selected based on maximizing the correlation between the estimated and the true contrast in the contrast–contrast parity plot. Specifically, for the low $\bar{k}$ data, we calculate the correlation of the 18 validation contrasts (corresponding to 18 validation datasets) relative to the ground truth contrasts. For the full-range $\bar{k}$ analysis, we use the average correlation from the contrasts from the three different $\bar{k}$ ranges [(0.025, 0.2), (1.0, 2.0), and (0.025, 2.0)]. An example of a sample validation plot is shown in Appendix A. In addition to the correlation, the validation and training loss functions were also monitored during the course of training. A sample plot for the full-range $\bar{k}$ data is also shown in Appendix A.

Here, again, it is worth emphasizing that the metric used to evaluate the photonizing task is important. For example, the overall accuracy is not necessarily a good metric since many photon maps have a small number of photons. Therefore, a model which uniformly predicts 0 for each pixel will show an uninformatively high accuracy, which is clearly not the desired performance and will lead to poor statistics. Similar issues have been documented in problems with high class imbalances,^42^ and correlation-based similarity metrics for evaluation are recommended therein.^43^ Since our final goal is to obtain a good estimate of the contrast, it is useful to use this information directly in the evaluation metric.

Finally, to obtain an estimate of the contrast from the predicted photon maps we use the maximum likelihood estimation (MLE) procedure to estimate the parameters of the negative binomial distribution. In general, the negative binomial distribution is a function of both C(q,t) and $\bar{k}$. However, we directly use a per-image estimate for $\bar{k}$ and therefore the MLE procedure reduces to a 1D optimization in C(q,t).^44^ We opt for the sequential 1D optimization procedure since, although the negative binomial distribution is parameterized by both $\bar{k}$ and the contrast, in practice the estimate of $\bar{k}$ is essentially converged and has almost no experimental uncertainty for the $\bar{k}$ ranges considered in this work.

## III. RESULTS AND ANALYSIS

### A. Accuracy

In this section, we compare the prediction quality of the CNN algorithms (both low $\bar{k}$ and full-range $\bar{k}$ models) against the GGG algorithm on data which simulates LCLS experiments and for which the ground truth contrast is known.^27^ The low count-rate data [ $\bar{k}$ ∈ (0.025, 0.2)] is analyzed using the low $\bar{k}$ model and the full-range data [ $\bar{k}$ ∈ (0.025, 2.0)] is analyzed using the full-range $\bar{k}$ model. To quantify performance, we show parity plots for the predicted and true contrast on datasets with varying contrasts. The error bars are derived from the reciprocal of the Fisher information, evaluated at the contrast estimate [ $I{\left(\hat{\beta }\right)}^{-1}$]. Thus, the 95% CI interval follows from assumed asymptotic normality of the MLE estimate. One crucial point is that these error bars quantify only the statistical error from fitting the negative binomial distribution and assume that the predicted photon maps are perfect (e.g., no ML or GGG bias). See Sec. III B for a more realistic estimate of the error, which accounts for both model bias and statistical uncertainty.

**FIG. 3.**
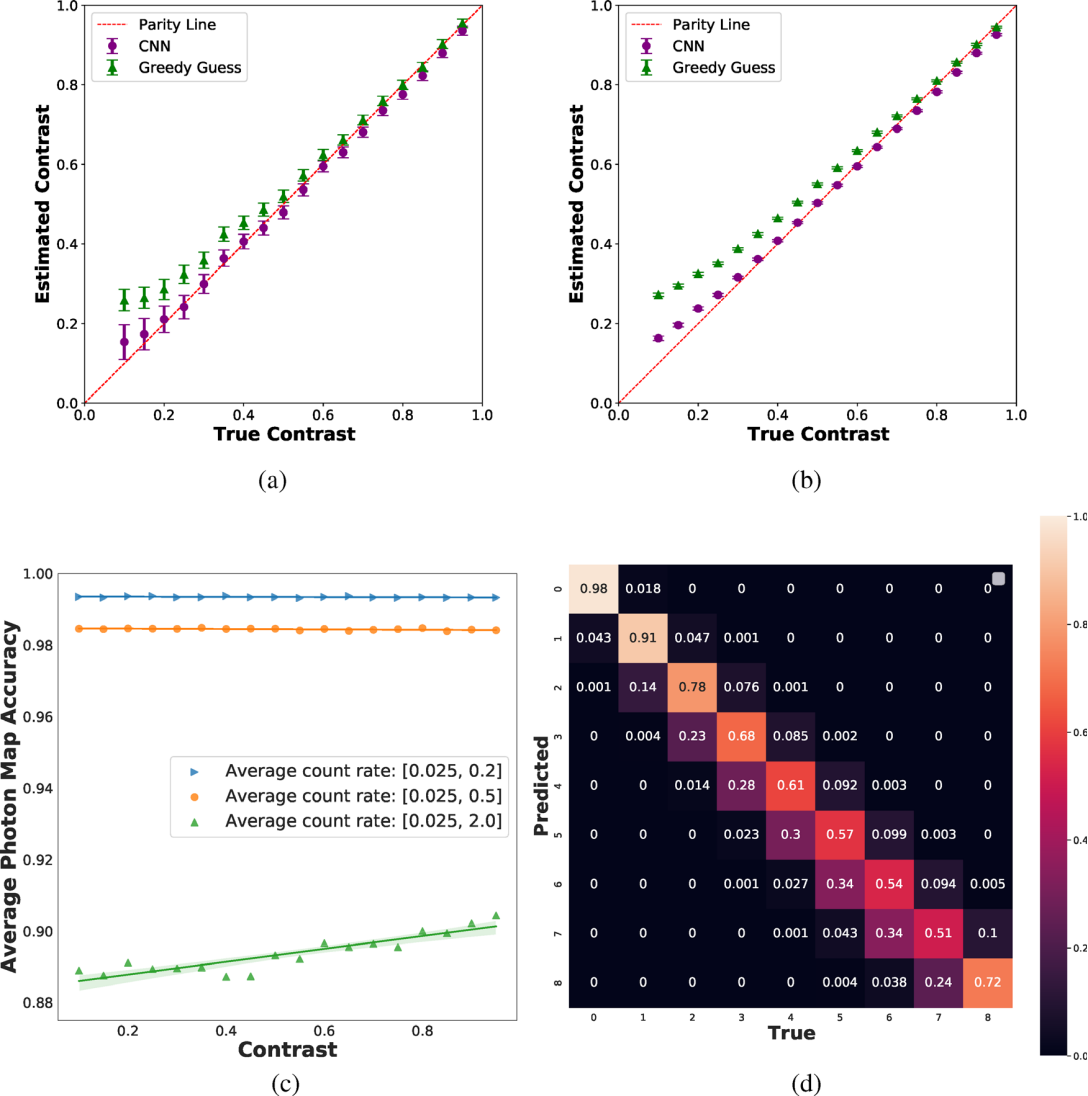
Predicted vs true contrast for CNN and GGG algorithms for (a) $\bar{k}$ ∈ (0.025, 0.2) using the low $\bar{k}$ model and (b) $\bar{k}$ ∈ (0.025, 2.0) using the full-range $\bar{k}$ model. Each data point corresponds to prediction on a test dataset of 2000 data points and subsequent maximum likelihood estimation (MLE). The CNN model exhibits smaller bias than the GGG algorithm at low contrasts and for the full-range $\bar{k}$ data. Error bars are obtained using the confidence interval based on the Fisher information. The error bars for the full $\bar{k}$ range are smaller than expected as imperfect photonization is not considered. See Sec. III B for a discussion of model uncertainty. (c) Average accuracy as a function of contrast level for three separate $\bar{k}$ ranges using the full-range $\bar{k}$ model. Error bars are obtained from the standard error in the slope of the linear regression fit. (d) Confusion matrices for full-range $\bar{k}$ model predictions on testing data with $\bar{k}$ ∈ (0.025–2.0). The model asymmetrically underpredicts high-photon events.

For low $\bar{k}$ and full-range $\bar{k}$ data at high contrasts, the CNN models and GGG algorithm give good predictions for the contrast. However, it is worth pointing out that in this regime the CNNs systematically underpredict the contrast and have slightly larger bias than the GGG algorithm. However, at low contrast levels, the GGG algorithm exhibits much greater bias than the CNNs [Figs. 3(a) and 3(b)]. One possible reason for the overall superior performance of the CNN models is that variation in photon charge cloud sizes and probabilities are accounted for in training. We note that even after optimizing droplet parameters on simulated data with known detector parameters, the GGG algorithm still exhibits large bias for lower contrast values, indicating that the algorithm may not have the complexity required to fully treat such data.

We also studied the performance of the full-range $\bar{k}$ model as a function of $\bar{k}$ and found that, on an average photon map accuracy basis, the prediction quality decreases with increasing $\bar{k}$ [Fig. 3(c)]. To further examine model errors, we clipped the output photon map to the range of (0, 8) (i.e., no photon map has more than eight photons or less than zero photons) and analyzed the confusion matrix (CF_*i*,*j*_) of the predictions [Fig. 3(d)]. The diagonal of the confusion matrix represents per-class accuracy. For instance, CF_2,2_ represents the accuracy of prediction for pixels containing two photons. It is evident that the model makes a greater proportion of errors for higher photon counts; note the trend does not hold for eight photon events due to the clipping operation. The off-diagonals of the confusion matrix indicate how the model makes errors. For example, the CF_3,6_ term indicates the probability of the model assigning three photons to a pixel when the true number of photons was actually equal to six. From these elements, we see that the CNN tends to underpredict high-photon events. These observations suggest dataset imbalance issues due to the fact that low-photon events are more probable in the training set (see Appendix C).

Although the full-range $\bar{k}$ model is less accurate on a per-photon map basis (relative to the low $\bar{k}$ model on low $\bar{k}$ data), this does not imply inferior contrast predictions. In fact, the parity plots are similar for low and full-range $\bar{k}$ cases (Fig. 3). This observation stems from the trade-off between information content and accuracy when higher $\bar{k}$ data are included (see Appendix B).

**FIG. 4.**
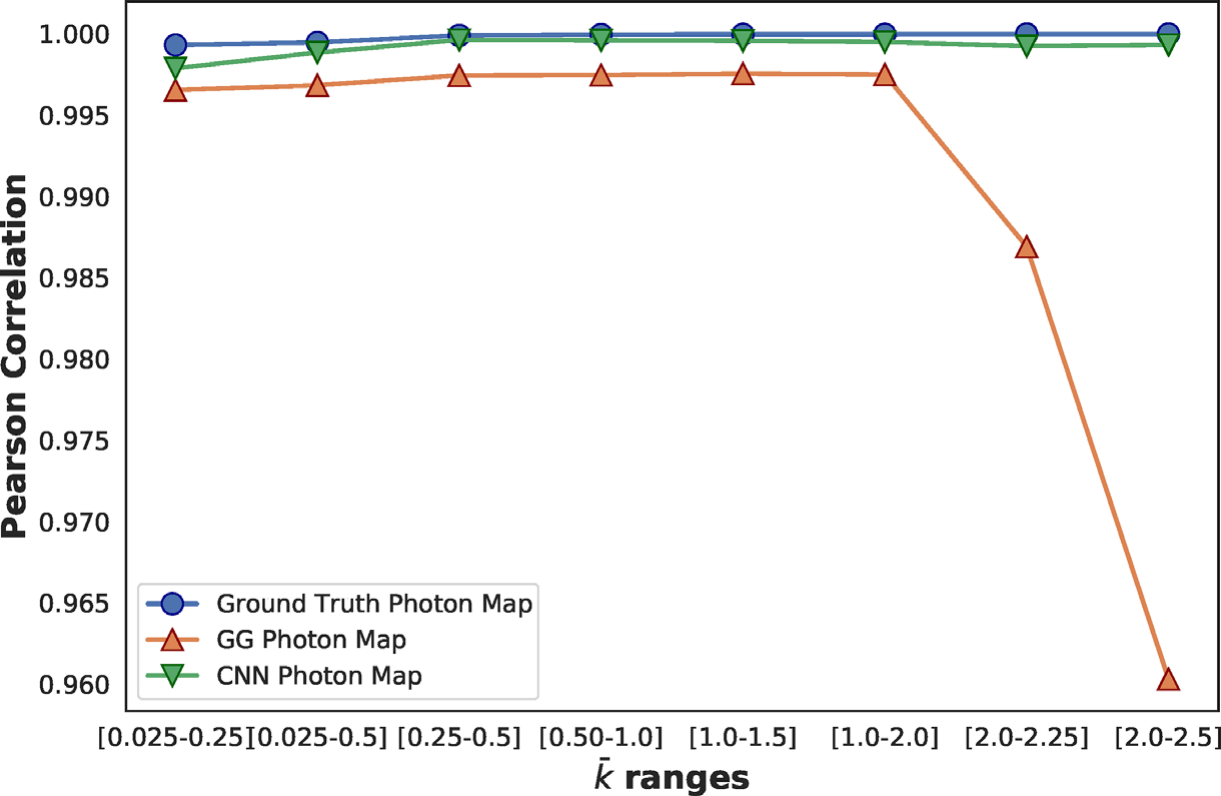
Contrast–contrast parity correlation for datasets generated using different $\bar{k}$ ranges. Here, the full-range $\bar{k}$ model was used for all predictions. Note, the ground truth photon maps do not have perfect correlation due to finite sampling statistics.

In Fig. 4, we quantify the performance of the full-range $\bar{k}$ model and the GGG algorithm at different $\bar{k}$ ranges using the correlation in the contrast–contrast parity plot as our metric. It is evident that the GGG algorithm is slightly biased across all $\bar{k}$ levels and performs poorly for $\bar{k}>2.0$. This is unsurprising, as droplet-based algorithms were designed to cope with small droplets with relatively few overlapping charge clouds. Furthermore, this implies further development of the CNN algorithm will be capable of handling datasets with large variation in $\bar{k}$ ranges.

Finally, one important concern is to consider the sensitivity of the CNN model to data from outside the simulated detector parameter distribution. Although the full-range $\bar{k}$ model clearly outperforms the GGG model based on the dataset shown in this manuscript, we cannot immediately exclude the possibility that the GGG may generalize better to a dataset with different simulation parameters. For this reason, we compared the performance of the full-range $\bar{k}$ model and the GGG algorithm on five datasets which are outside the training detector parameter distribution and found that the CNN models were significantly more robust. This analysis is detailed in Appendix D.

### B. Uncertainty quantification

In this section, we consider a neural network ensemble approach to quantify the uncertainty in the predicted photon maps and contrasts. The motivation for such an analysis is that, while deep learning models have exhibited significant successes in their application to scientific problems, they have a tendency to engender overconfident predictions that may be inexact. As an example, neural networks are unable to recognize Out Of Distribution (OOD) instances and habitually make erroneous predictions for such cases with high confidence.^45–47^ In reliability-critical tasks, such errors and uncertainties in model predictions have led to undesirable outcomes.^48,49^ In this context, quantifying the uncertainties in deep learning model predictions is highly desirable.

There are two sources of predictive uncertainty that need to be considered: Epistemic and Aleatoric. Epistemic uncertainty^50^ (reducible or subjective uncertainty) arises due to lack of knowledge regarding the dynamics of the system under consideration, or an inability to express the underlying dynamics accurately using models. Epistemic uncertainties can lead to biases in the predictions. Aleatoric uncertainty^50^ (irreducible uncertainty or stochastic uncertainty) arises due to noise in the training data, projection of data onto a lower space, absence of important features, etc. Aleatoric sources can lead to variances in the predictions.

For our analysis, we use an ensemble of neural networks to make a point prediction of the contrast C(q,t) as well as to give an estimate of the predicted uncertainty. We train ensembles, denoted low $\bar{k}$ ensemble and full-range $\bar{k}$ ensemble, which correspond to the same data used to train the low and full-range $\bar{k}$ models. This is in line with model ensembling based uncertainty quantification (UQ) methods validated in literature.^51,52^ Such ensembling accounts for aleatoric uncertainties due to the data and weight uncertainties. In our investigation, the neural network ensemble is formed via sequential sampling, wherein ten partially decorrelated models were sampled during the model training. Contiguous samples were spaced by ten optimization epochs each. Here it is pertinent to note that we investigated other uncertainty quantification methods including Monte Carlo dropout,^53^ deep evidential regression^39^ and Probabilistic Neural Networks (PNNs that utilize the full negative log likelihood without assuming homoscedasticity). We found that the PNN and deep evidential approach had very poor convergence for our problem in our experiments, and that the Monte Carlo dropout scheme did not engender calibrated prediction intervals on a held-out validation set. Therefore, in our experiments, the ensemble-based approach gave the best point prediction and uncertainty estimate on a held-out validation set. Here, we emphasize that our experimentation on various uncertainty quantification approaches is not exhaustive, and we envision substantial future work in this area.

**FIG. 5.**
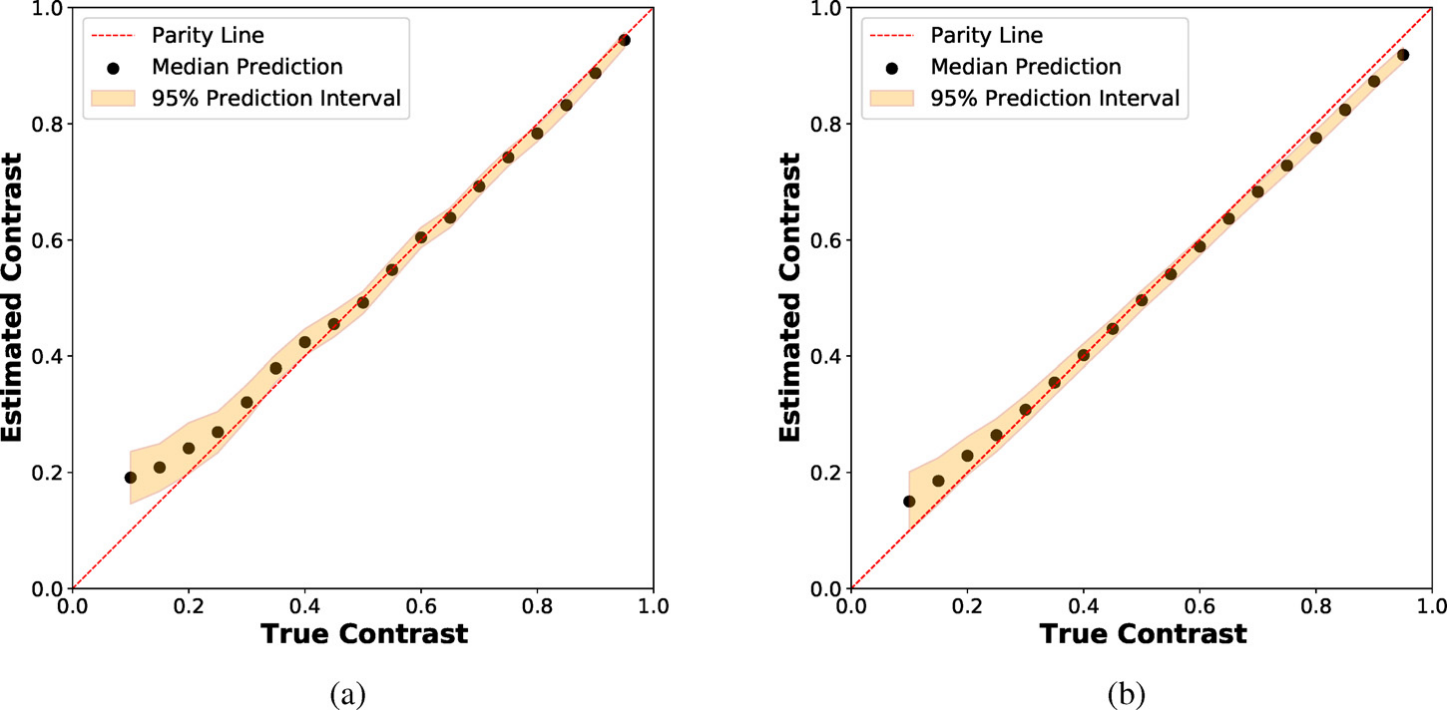
Contrast–contrast parity plot for data in the $\bar{k}$ range of (a) (0.025, 0.2) using the low $\bar{k}$ ensemble and (b) (0.025, 2.0) using the full-range $\bar{k}$ ensemble. The contrast point prediction is obtained from the median contrast prediction and the 95% prediction intervals follows from assumed *t*-distribution statistics.

For the ensemble, the contrast is calculated for each model via a maximum likelihood procedure and the contrast point prediction is taken as the median predicted value. To estimate the model uncertainty, we provide a 95% contrast prediction interval using the standard deviation of the predicted contrasts and making the assuming that the predictive distribution follows a *t*-distribution with nine degrees of freedom (Fig. 5).

We see that the error bars are larger at lower contrasts, which correctly captures the notion that the prediction task is harder at lower contrasts.^27^ We also notice a systematic bias in the CNN models at high contrasts. This bias may arise due to the epistemic uncertainties due to the model form (structural uncertainty). Such structural uncertainties in deep learning models cannot be accounted for by any extant approach, including the procedure used in this investigation. However, this indicates a need for more refinement on the present approach (for instance, more fine grained optimization of the model architecture, etc.) and will be explored more fully in future work.

**FIG. 6.**
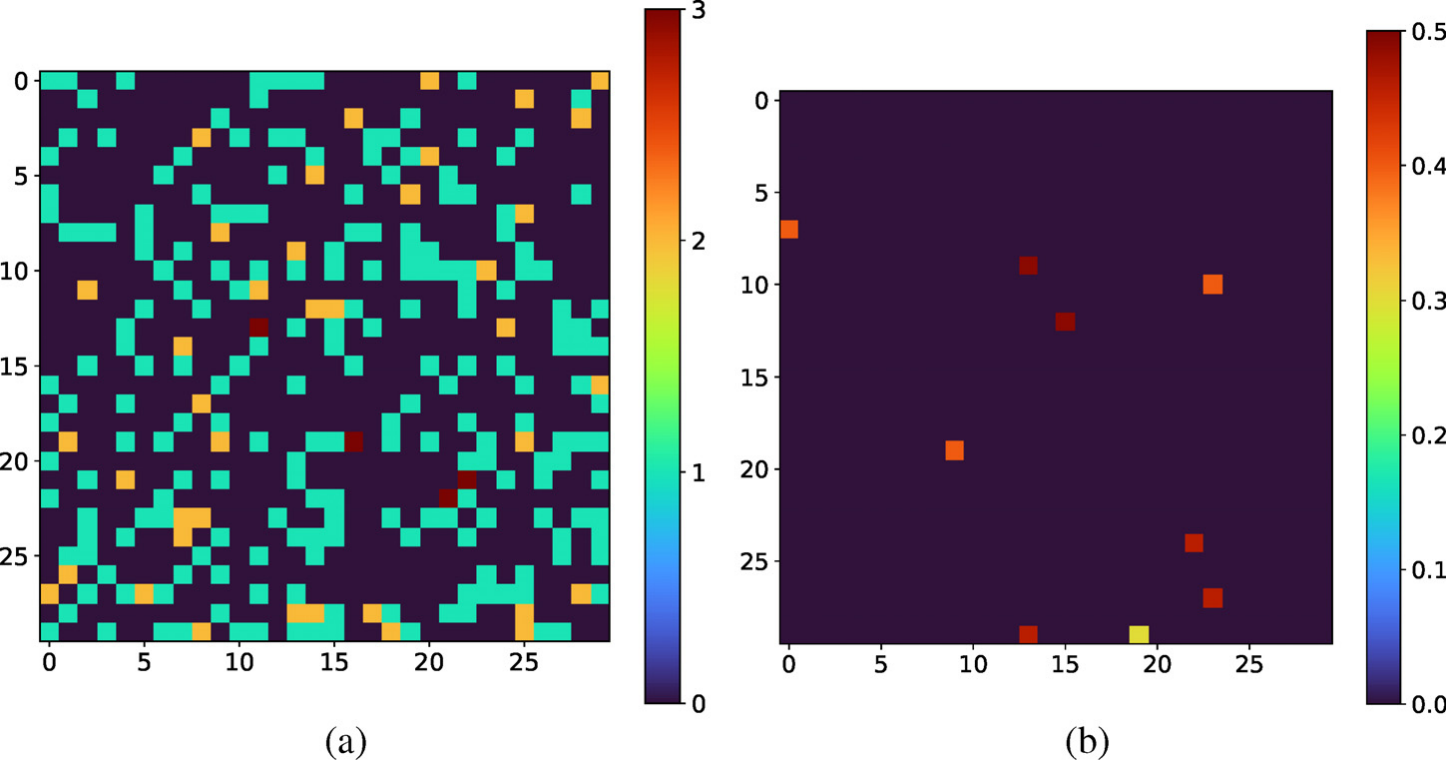
(a) The full-range $\bar{k}$ CNN ensemble used to visualize the (a) median prediction photon map and (b) the standard deviation prediction on a sample photon map drawn from data in the range (0.025, 2.0) $\bar{k}$. This analysis allows users to visualize the region of the image where the photon assignment may be challenging.

Another interesting avenue is to consider the median predicted photon map and the predicted standard deviation map to examine where the neural networks lack consensus. An example of this pair of outputs are shown in Fig. 6. Evidently, the ensemble predicts insignificant uncertainty for the majority of the image with the exception of a few pixels with relatively high uncertainty. An interesting future strategy could involve using the CNN model as a fast, initial approach and subsequently run more complex fitting algorithms on regions of the image with high predicted uncertainty.

### C. Speed of inference

As x-ray sources and detectors move toward faster repetition rates nearing 1 MHz, it is important to preserve the possibility of live data analysis. Here, we compare the speed of an optimized GGG droplet algorithm^27^ against the trained full-range $\bar{k}$ CNN model (see Table II). On 1 CPU, the CNN outperforms the GGG algorithm by roughly an order of magnitude. This advantage stretches to two orders of magnitude when comparing GGG parallelized across multiple CPU cores to the CNN running on one NVIDIA A100 GPU.

**TABLE II.** Speed comparison between the full-range $\bar{k}$ CNN model and the GGG algorithm. Rates are reported for a prediction on 1000 XPFS shots. Using a CNN deployed on GPU hardware yields a speedup of two orders of magnitude relative to a multi-CPU droplet implementation.

Device	Algorithm	Rate (kHz)	Rate relative to 1 CPU
1 CPU	GGG	0.008	1
12 CPU	GGG	0.05	6
32 CPU	GGG	0.1	12
1 CPU	CNN	0.2	27
1 GPU	CNN	5.0	700

**FIG. 7.**
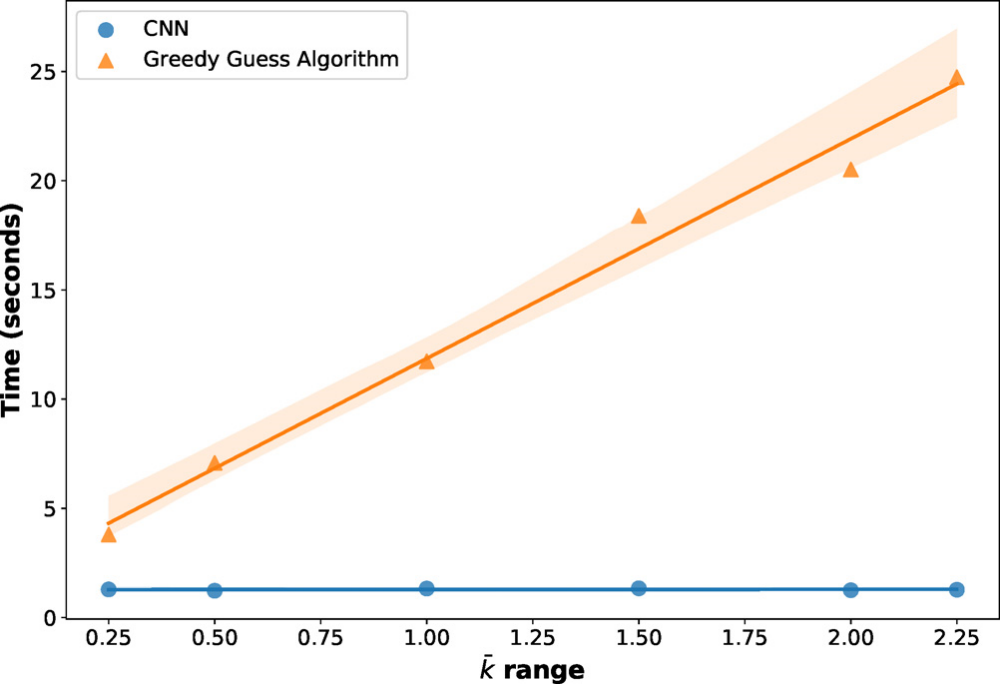
Time to make predictions on 2000 XPFS frames for the full-range $\bar{k}$ CNN model vs the GGG algorithm as a function of $\bar{k}$. Error bars are obtained from the standard error in the slope of the linear regression fit. The CNN exhibits constant scaling with $\bar{k}$ while the GGG scaling is observed to be linear.

The observed speedup presented here is consistent with intuition. At inference, the trained neural network, which consists primarily of matrix multiplication operations, is efficiently parallelized over thousands of GPU processes.^54^ In contrast, the GGG algorithm requires for-loop operations at the level of each droplet. For this reason, one additional beneficial property of the CNN model is that the prediction rate does not depend on the content of the XPFS frames and is consequently independent of $\bar{k}$. In contrast, for the GGG algorithm, the run time scales linearly with $\bar{k}$ (Fig. 7). Here, it is worth mentioning that the GGG algorithm is already orders of magnitudes faster than the droplet least squares algorithm,^24^ which is exponential in computational complexity. Such advances in speed will be important in future experimentation for tasks such as live-contrast monitoring as well as deciphering, in real-time, the number of frames needed to give sufficient accuracy for a contrast measurement.

**FIG. 8.**
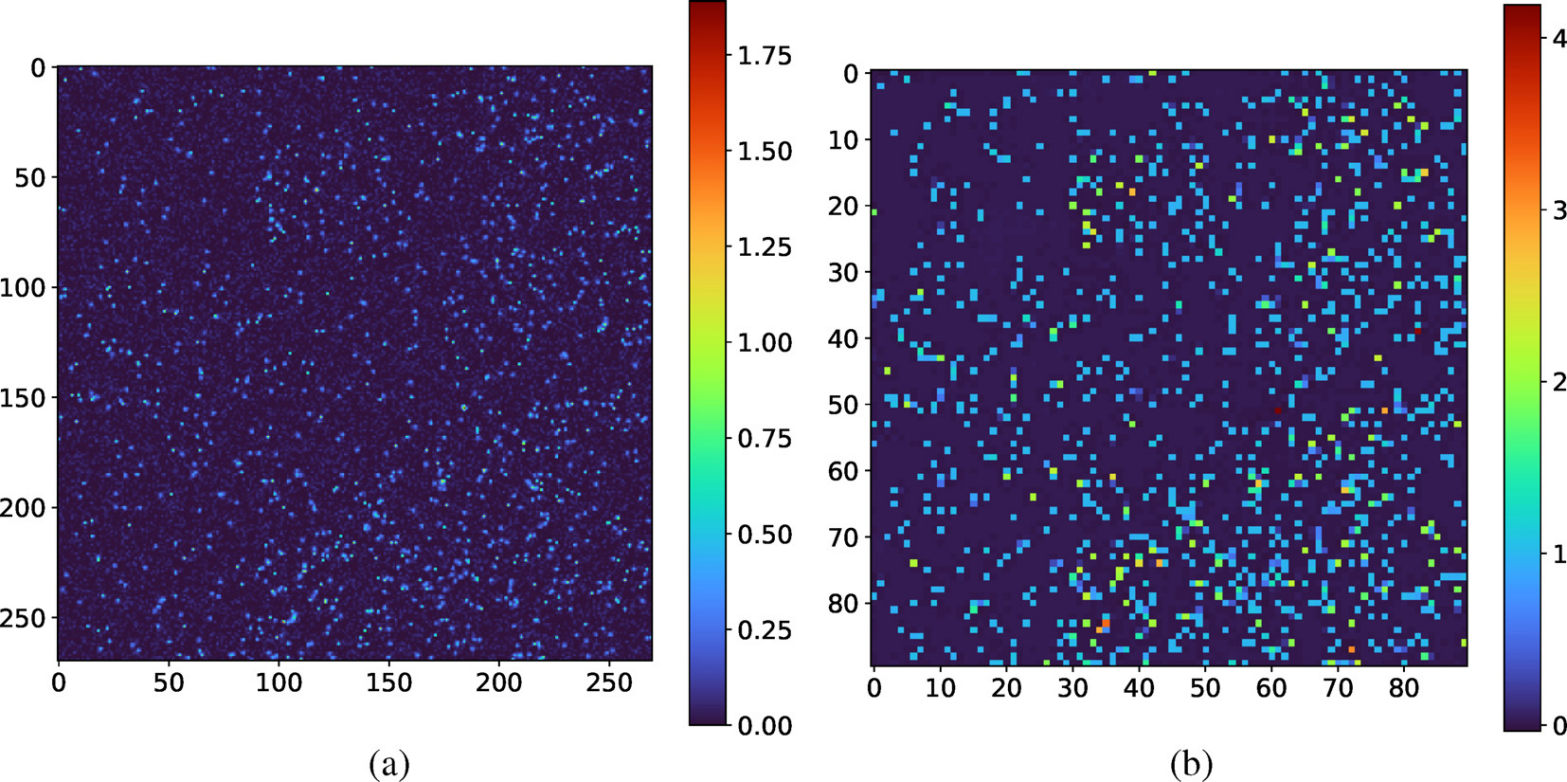
(a) A larger input detector image with 270 × 270 pixels and (b) corresponding predicted photon map (90 × 90 pixels). The full-range $\bar{k}$ CNN model is able to make predictions on larger inputs than it was trained on.

Finally, since the neural network architecture follows a fully convolutional paradigm, it is possible to make predictions on larger input/detector sizes than those used in the training set. This is enabled by the fact that fully-convolutional architectures learn local spatial filters which apply to the full image, making training relatively efficient. For this analysis, the neural network can handle input sizes of (*N_f_*, 90*a*, 90*b*, 1), where *a*, *b* are positive integers and *N_f_* denotes a variable number of frames. Note, that this allows for the model to be used on any detector size after zero-padding to the nearest (90*a*, 90*b*) frame size and does not require additional layers for a sliding window approach. We calculate the average time to make predictions on datasets of dimensionality (100, 90, 90, 1), (100, 270, 270, 1), and (100, 900, 900, 1). We observe rates of 3.4, 3.1, and 0.3 kHz, respectively. As the rate only decreases a factor of 10 between a frame size of (90, 90) and (900, 900), it appears that we do not observe quadratic scaling that would be observed using droplet-based algorithms. Furthermore, the ability to analyze such data in a single-shot manner is a significant advantage over the sliding approach for droplet analysis which has been developed.^33^ A representative example of the CNN prediction using an input resolution of 270 × 270 pixels is shown in Fig. 8.

## IV. CONCLUSIONS AND FUTURE OUTLOOK

In this work, we have developed a convolutional neural network architecture that is capable of analyzing single-photon x-ray speckle data in non-optimal situations, such as for small pixel size detectors or with soft x-ray energies. We have benchmarked this algorithm on realistic simulated data and found that it outperforms the conventional Gaussian Greedy Guess (GGG) droplet algorithm in terms of speed and computational complexity. Furthermore, the algorithm is able to extract the contrast information for new ranges that were previously inaccessible, such as under low contrast conditions—relevant for systems which scatter weakly, as well as in a high $\bar{k}$ regime.

An important area of future work is to integrate the neural network into a live analysis pipeline. Here, one challenge will be to develop software pipelines capable of monitoring and fitting detector parameters over the course of the experiment. Then, if the detector parameters diverge significantly from that of the training set, new data should be automatically simulated and the models should be accordingly updated. These considerations affect both the CNN and GGG and therefore live detector parameter monitoring will be a generically welcome development for the future. For the CNN, this approach would likely be combined with methods such as online learning^55^ or transfer learning^56^ in order to avoid re-training models from scratch.

Such developments will create new opportunities to perform live studies of fluctuations using XPFS in novel systems, such as in quantum or topological materials.

## Figures and Tables

**FIG. 9.**
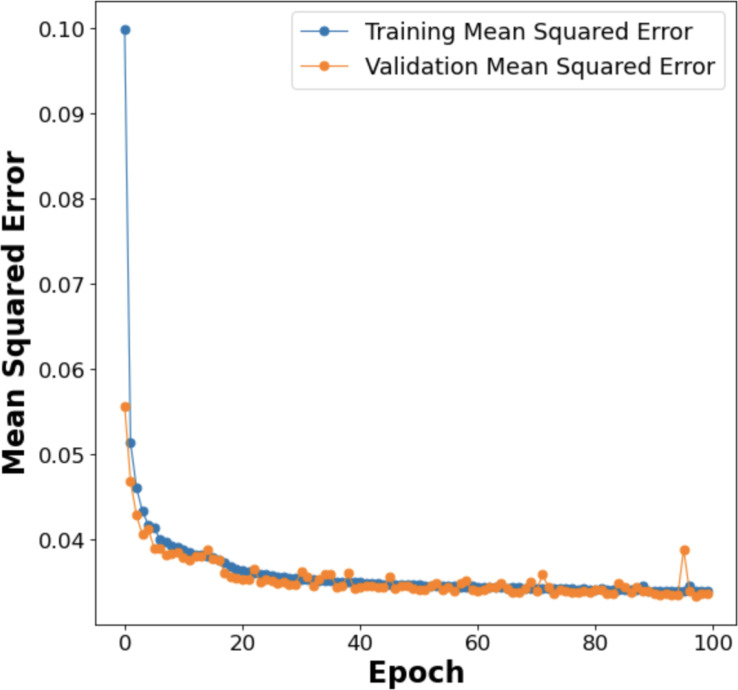
Sample plot showing the mean-squared-error as a function of epoch number for validation and training data for the full-range model.

**FIG. 10.**
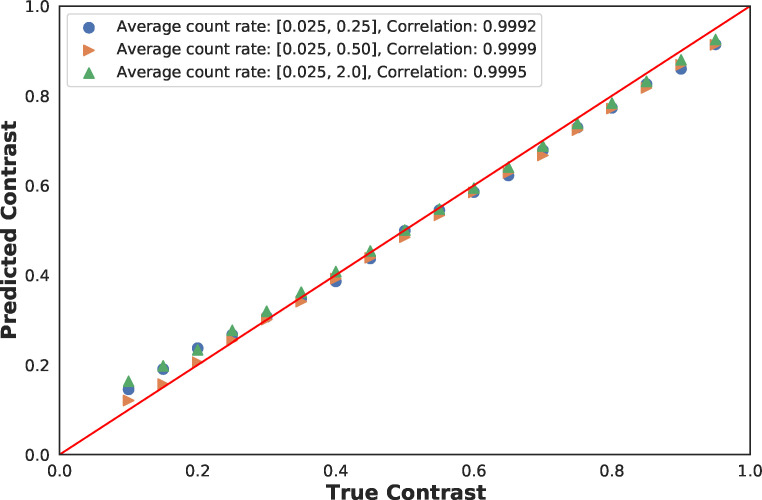
Correlation in contrast–contrast parity plot. Here, each data point represents the predicted contrast over 5000 frames after fitting to negative binomial statistics. The overall evaluation metric of a model is the average of the correlations for the different $\bar{k}$ ranges. In this instance, the average correlation is 0.9989.

**FIG. 11.**
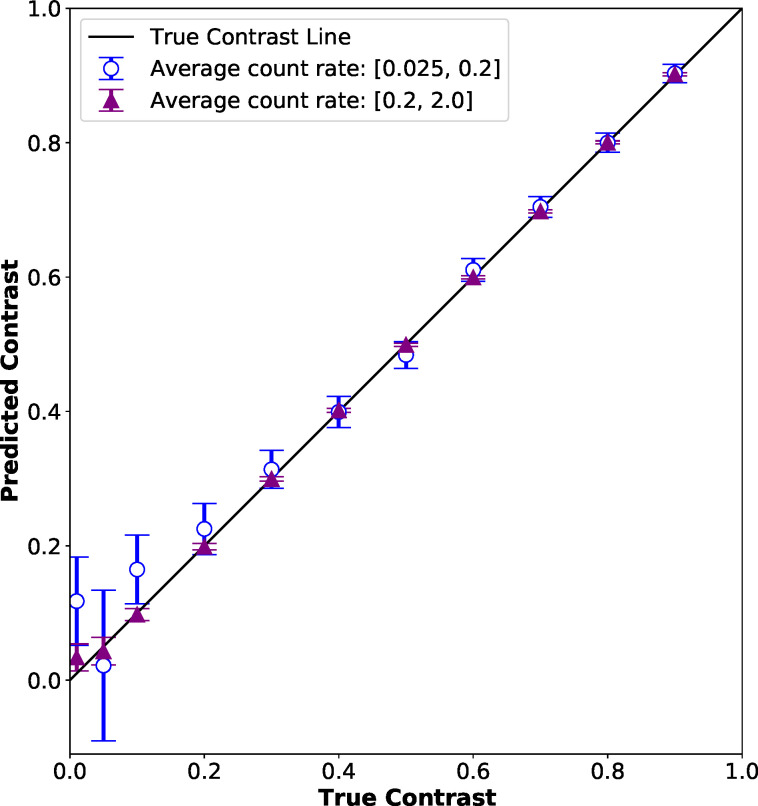
Comparison of fitting for high $\bar{k}$ data vs low $\bar{k}$ on data with perfect photon maps (no error from denoising). Evidently, the error shrinks for higher $\bar{k}$ data.

**FIG. 12.**
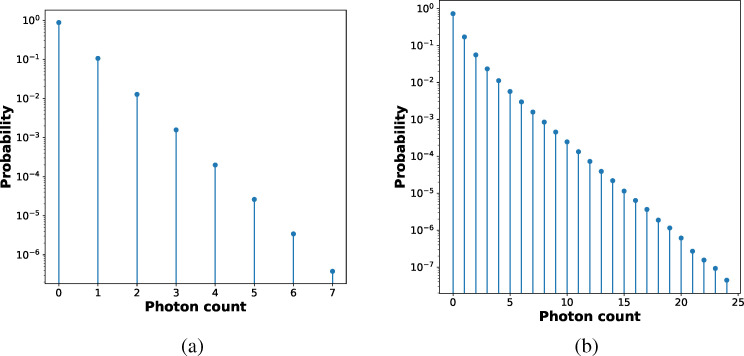
Photon count probability distributions for (a) $\bar{k}$ in range (0.025, 0.2) and (b) $\bar{k}$ in range (0.025, 2.0).

**FIG. 13.**
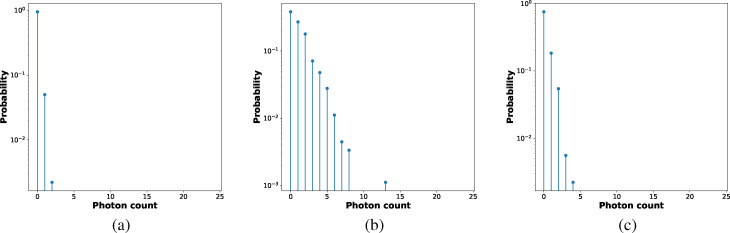
Photon count probability distributions for three different images (per-frame) sampled from $\bar{k}$ in range (0.025, 2.0).

**FIG. 14.**
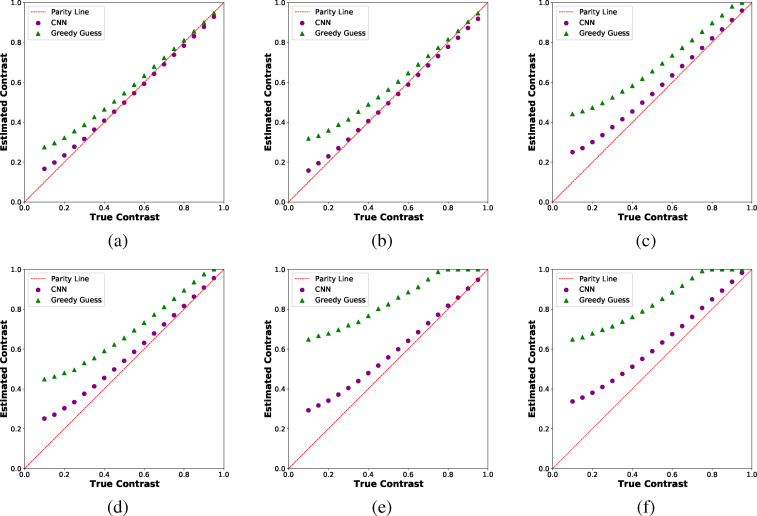
Comparison between the ML and GGG algorithms for simulation parameters outside of the training simulator parameter distribution. The specific conditions in (a)–(f) are detailed in Table III. For this analysis, the full-range $\bar{k}$ model was used to make predictions.

## Data Availability

The data that support the findings of this study are openly available at https://doi.org/10.5281/zenodo.6643622, Ref. 57. Code is freely available at https://github.com/slaclab/ml_xpfs.

## APPENDIX A: MODEL TRAINING AND VALIDATION METRICS

The training and validation mean-squared-error loss functions were monitored as a function of epoch number in order to assess whether the model exhibits overfitting. Figure 9 shows a representative loss function plot corresponding to the model trained for the full range of data (0.025, 2.0). Here, it is clear that the loss function values are similar between training and testing and, notably, that the validation loss continues to decrease even after 100 epochs.

In addition, since the contrast is the important metric in this analysis, we also opt to use the average correlation (across datasets with different average count-rates) of the contrast–contrast parity model as a metric for model validation. This metric can be visualized in Fig. 10.

## APPENDIX B: VALUE OF HIGH $\bar{k}$

Here, we consider the value of utilizing data at higher $\bar{k}$ in the fitting process. In this scenario, the photon maps are perfect samples from the negative binomial distribution. In other words, the models developed in this paper are not used to generate the photon maps and therefore there is no error owing to detector image to photon map conversion. At higher $\bar{k}$, high-count photon events are more probable and contain more information about the underlying distribution relative to common events (e.g., 0 or 1 photon events). It follows naturally that incorporating higher $\bar{k}$ data allows for fitting the distribution with less data and more accuracy relative to only low $\bar{k}$ fits. In Fig. 11, we show the results of fitting the negative binomial distribution with $\bar{k}$ varying uniformly in two ranges, (0.025, 0.2) and (0.2, 2.0) for 2000 data points. We see that for the higher $\bar{k}$ range, the error bars in the contrast are much smaller relative low $\bar{k}$.

Note that the argument made in this section is purely of a statistical nature based on the underlying negative binomial distribution and with no use of any model (ML or GGG). In practice, and as shown in Fig. 3, model performance is worse at higher average photon count. Therefore, there exists a trade-off between model accuracy and value of information.

## APPENDIX C: PHOTON TRAINING SET DISTRIBUTIONS

In this section, we show the overall photon count distributions for the low $\bar{k}$ and full-range $\bar{k}$ datasets [Figs. 12(a) and 12(b)]. These distributions show that there is a definite dataset imbalance due to over-representation of low-photon events.

On a per-frame basis, the probability distributions vary because each training frame samples a different $\bar{k}$ and contrast. Three such individual photon count distributions are shown in Fig. 13.

## APPENDIX D: SENSITIVITY ANALYSIS

In this section, we consider the robustness of the machine learning model to different choices of detector simulation parameters. Specifically, the choices of simulation parameters represent extrapolation and interpolation of those parameters presented in the main text. We varied three specific parameters: the baseline noise term, the charge cloud sizes and their respective probabilities. The variable simulation parameters are delineated in Table III. For this analysis, the simulated testing data comes from the (0.025, 2.0) $\bar{k}$ range.

**TABLE III.** Simulation parameters for various experiments used to test the sensitivity and robustness of the ML and GGG models.

Simulation	Charge cloud size (*σ_G_*)	Probabilities (*w_i_*)	Background noise (*σ_N_*)
(a)	(0.1, 0.25, 0.35, 0.45, 0.55, 0.6)	(0.25, 0.15, 0.1, 0.3, 0.15, 0.05)	15
(b)	(0.1, 0.25, 0.35, 0.45, 0.55, 0.6)	(0.166, 0.166, 0.166, 0.166, 0.166, 0.166)	15
(c)	(0.05, 0.15, 0.2, 0.4, 0.7, 0.8)	(0.166, 0.166, 0.166, 0.166, 0.166, 0.166)	15
(d)	(0.05, 0.15, 0.2, 0.4, 0.7, 0.8)	(0.166, 0.166, 0.166, 0.166, 0.166, 0.166)	17
(e)	(0.025, 0.25, 0.5, 0.75, 0.9, 1.1)	(0.166, 0.166, 0.166, 0.166, 0.166, 0.166)	20
(f)	(0.01, 0.02, 0.03, 0.7, 1.0, 1.5)	(0.166, 0.166, 0.166, 0.166, 0.166, 0.166)	22

In simulation (a), we consider a control experiment with the same simulation parameters as reported in the main text. The performance of the full-range $\bar{k}$ ML model and GGG is very close to the dataset reported in Fig. 3. In (b), the baseline noise levels and the charge cloud sizes are kept the same and only the probability distribution over charge cloud sizes are changed. This scenario is a small perturbation which would expose the model to similar configurations in the training set, however, with different weighting. We do not observe any significant change for the ML model relative to the control. Conversely, the GGG prediction quality decreases. In (c), the model is forced to make a prediction on extrapolated and interpolated charge cloud sizes. For example, (0.05, 0.7, 0.8) are outside the range of simulated charge cloud sizes and (0.15, 0.2, 0.4) are within the range of previous simulations. The contrast parity plots corresponding to the different perturbation experiments are shown in Fig. 14. Here again both models' predictions deteriorate; however, the machine learning model is more resilient and still accurate at high contrast. In (d), the baseline noise is increased relative to (c). We note that a similar performance is observed relative to (c) for both models. Finally, (e) and (f) constitute conditions that are quite far out of distribution and much further than would be tolerated during an experiment. In a practical implementation, the model would likely need to be retrained under such conditions. However, interestingly, we find that the machine learning model still performs admirably in these conditions. On the other hand, the GGG model is not robust to these new parameters.
